# Measuring the Complexity of Self-Regulated Learning and Academic Challenges for Adolescents in Canada

**DOI:** 10.1177/07342829231221851

**Published:** 2023-12-15

**Authors:** Meng Qi Wu, Violet V. Cieslik, Safoura Askari, Allyson F. Hadwin, Moira Hood

**Affiliations:** 1 Department of Educational Psychology and Leadership Studies, 223046 University of Victoria, Victoria, BC, Canada; 2 Department of Psychology, 175081 University of Victoria, Victoria, BC, Canada

**Keywords:** secondary education/adolescence, social and educational environment, measurement, student engagement, education program and leadership, self-regulated learning, academic challenges

## Abstract

Research that uses self-report measures to examine the complexity of self-regulated learning (SRL) and academic challenges for adolescents is limited. This study examined the psychometric property of the Self-Regulated Learning Profile and Self-Diagnostic (SRL-PSD) instrument and addressed the multi-components of SRL and academic challenges for adolescents. Participants were 358 adolescents from a Canadian middle school. The subscales of SRL-PSD were administered to students through LimeSurvey during a 25-min instructional session over two days. Results demonstrated the SRL-PSD was a reliable and valid self-report instrument to measure adolescents’ SRL practices and academic challenges. Also, all types of SRL practices and academic challenges were significantly intercorrelated. Additionally, all types of SRL practices were positively associated with school engagement, whereas all types of academic challenges were negatively associated with school engagement. Overall, this study provides a validated self-report measure for educators and researchers to examine adolescents’ SRL practices and academic challenges.

## Introduction

### Self-Regulated Learning for Adolescents

During the transition from elementary education to secondary education, adolescents are expected to become independent and manage different academic tasks and challenges from multiple disciplines (Schunk & Miller, 2002; Vandevelde et al., 2013). However, the unsynchronized development of cognitive control and motivational regulation during adolescence can lead to heightened vulnerability to risk-taking behaviors and challenges (Smith et al., 2013). Self-regulated learning (SRL) is essential for learners to develop the lifelong learning skills needed to succeed in education and beyond (Hadwin & Winne, 2012; Theobald, 2021; Winne, 2015; Winne & Hadwin, 2008). Successfully adapting to academic environments requires regulated processes including strategically planning, monitoring, evaluating, and adapting to individual learning goals and situations (Schunk & Zimmerman, 1997; Zimmerman & Schunk, 2001). These regulatory processes in learning help students build strong metacognitive knowledge (Pintrich, 2000), which subsequently helps students to strategically manage their learning processes (Winne, 2015).

In addition, Winne and Hadwin’s (1998) SRL model considers SRL as an active, constructive process allowing learners to metacognitively monitor and regulate their cognition, decision-making, planning, motivation, affect, and emotion as well as factors in the external environment (Hadwin et al., 2022; Winne, 2015; Winne & Hadwin, 1998). This notion is in line with Pintrich’s (2000) and Vandevelde et al.’s (2013) acknowledgments of SRL comprising various components (e.g., cognition, motivation, metacognition, and emotion) that are essential for successful learning and the reciprocal and recurrent interactions between these components. Notwithstanding the importance of SRL, the developmental period of adolescence poses unique challenges for middle school students’ learning.

Adolescence is a time of gross developmental changes (i.e., neurobiological, cognitive, and social; Steinberg & Morris, 2001), which may lead to the lack of skills for effectively and strategically regulating their emotion, cognition, and behaviors (Smith et al., 2013; Steinberg, 2005). This inability to regulate may prevent adolescents from developing metacognitive knowledge about their academic tasks, choosing effective learning strategies, recognizing adaptive and maladaptive patterns, and making adaptations for their future learning. Even though existing studies have shown that engaging in SRL practices helps adolescents to take control of their learning (Kolovelonis et al., 2022), it is important to recognize that disengagement with these practices could influence their academic performance.

### Academic Challenges for Adolescents

Throughout adolescence, middle school students face neurobiological, social, and academic changes (La Greca & Harrison, 2005; Romero et al., 2014). Navigating these changes in adolescence might be difficult, as adolescents have not fully developed competency to effectively regulate their behaviors and emotional states. This unique characteristic of adolescents can further increase adolescents’ academic challenges. For example, time management and procrastination remain difficult for adolescents (Dembo & Eaton, 2000). Studies have demonstrated that changes in classroom settings and grading standards coupled with increased academic expectations have been associated with decreased motivation (Lerner & Steinberg., 2009) and engagement (Eccles et al., 1993; Skinner & Pitzer, 2011), and increased rates of dropout (Juvonen et al., 2004), lower grades (Anderson et al., 2000; Blackwell et al., 2007), and unfavorable attitudes toward academics in middle school populations (Lerner & Steinberg., 2009; Romero et al., 2014). Theoretically, academic challenges provide opportunities for learners to take control of their (meta)cognition, motivation, behavior, and emotion (Hadwin et al., 2022). Hadwin et al. (2022) found that academic challenges are strongly associated with SRL practices for university students. However, no existing research has examined the multifaceted nature of academic challenges for adolescents.

### Self-Regulated Learning and Academic Challenge Measure Among Adolescents

Existing SRL instruments do not capture the full spectrum of SRL (e.g., MSLQ, ASSIST, and MAI) as researchers tend to combine different SRL instruments to examine psychological constructs within SRL (Vandevelde et al., 2013). However, this approach lacks consideration of the psychometric properties of combined SRL instruments. To address these limitations, the present study validated the Self-Regulated Learning Profile and Self-Diagnostic (SRL-PSD) instrument (Hadwin et al., 2022) for adolescents. The SRL-PSD instrument includes SRL importance (SRL-I), SRL self-efficacy (SRL-SE), SRL practices, and academic challenges. Additionally, the design of the SRL-PSD instrument provides opportunities for learners to make connections between SRL practices and academic challenges.

Although the importance of SRL has been well established in young children and post-secondary students (e.g., Toering et al., 2012), less is known about adolescents transitioning from elementary to secondary school. Adolescents in secondary education may not know how to engage in SRL practices or select effective self-regulatory strategies (Butler et al., 2011), thereby resulting in more academic challenges and poorer academic performance. As no existing SRL measure has been developed to jointly assess all these components, this paper contributes to the SRL research by validating the SRL-PSD instrument for adolescents. Validating the measure is a critical step for future research to investigate the interactive relationship between SRL practices and academic challenges for this unique population.

Furthermore, research has demonstrated that academic engagement behaviors (e.g., participating in learning activities; Sinatra et al., 2015) reflect students’ underlying motivation for school (Skinner et al., 2009), which helps students develop task understanding and engage strategically in their learning. Therefore, it is essential to understand the relationships among SRL practices, academic challenges, and student engagement behaviors for adolescents. Additionally, school aversion defined as the inverse of emotional engagement has been found to be related to lower social connectedness and lower satisfaction with school (Lewis et al., 2018). Experiencing school aversion can lead to increased academic challenges and diminished academic performance. However, no existing study has evaluated the relationships among middle school students’ school aversion, engagement behaviors, SRL practices, and academic challenges. As a result, we evaluated the concurrent validity of the SRL Practices and Challenges subscales in terms of their associations with school engagement and feelings toward school.

## Methods

### Participants

Participants were 358 adolescents from a middle school located in the Sooke School District of the province of British Columbia (BC) in Canada. In our sample, there were 139 students in grade six, 97 students in grade seven, and 90 students in grade eight. Grade was unspecified for 32 students who did not respond to the demographic question. The sample included 159 female students, 152 male students, five non-binary students, one two-spirit student, 14 students preferred not to say, and 27 students who did not provide gender information.

### Procedure

The research objectives were discussed at school, and students had the chance to withdraw from the study at any stage of the research. Details about the research component, instructional unit, and consent options were given to parents and guardians. Questionnaires were administered through LimeSurvey. Flexibility was allowed if a student missed a day of school and was able to complete questionnaire items within the allotted 2-week window, as per COVID norms for classroom teaching. Teachers from grades 6 to 8 implemented a 25-min lesson with various topics, such as school engagement, SRL practices and challenges, and COVID-19-related stress for five school days during a school week. After completing the questionnaires, students were provided with activities to reflect, self-assess, and evaluate their approaches to learning.

### Measures

#### Self-Regulated Learning

The SRL-Practices Scale (SRL-P; Hadwin et al., 2021) measures students’ perceptions about their engagement in practices that foster SRL. The SRL-P comprises 31 items yielding eight subscales related to (1) task understanding (e.g., “I considered what knowledge or big ideas I should learn or demonstrate”; 5 items), (2) goal management (e.g., “I set goals that would be useful for checking my progress”; 5 items), (3) motivation appraisal (e.g., “I assessed if I think I can do it”; 3 items), (4) task value (e.g., “I thought about why we are being asked to know this”; 3 items), (5) time management (e.g., “I check to see if I was staying on time”; 3 items), (6) monitoring (e.g., “I asked myself if I understand what I am supposed to be doing”; 3 items), (7) adapting (e.g., “I altered the level of effort I put in”; 6 items), and (8) socioemotional engagement (e.g., “I had fun in class discussions and activities with my teacher or my classmates”; 3 items). Participants responded to each item on a five-point Likert scale (1 = *strongly disagree* to 5 = *strongly agree*). Higher scores indicate increased engagement with SRL practices.

#### Academic Challenges

Academic Challenges Scale (Hadwin et al., 2021) comprises 31 items with six subscales reflecting the degree to which students encountered challenges during their learning, including motivational challenge (e.g., “Feeling like my work was worth doing”; 3 items), (2) metacognitive challenge (e.g., “Knowing how or when to fix strategies/study skills”; 9 items), (3) initiating and sustaining challenge (e.g., “Wanting to do my schoolwork”; 4 items), (4) cognitive challenge (e.g., “Understanding learning materials and concepts”; 6 items), (5) goal and time management challenge (e.g., “Keeping my commitments or goals”; 4 items), and (6) social and emotional challenge (e.g., “Taking care of my mental health and well-being”; 6 items). Participants responded to each item on a five-point Likert scale (1 = *strongly disagree* to 5 = *strongly agree*). Higher scores indicate greater levels of struggles to manage aspects of studying.

#### School Engagement

The New Zealand Council for Educational Researcher (NZCER)’s Me and My School survey (see Darr, 2012) was used. This study selected nine items from the cognitive and behavioral subscales of the Me and My School survey (e.g., “*I pay attention in class*” and “*I look for ways to improve my schoolwork*”). Priority was given to items that were relevant in the middle school context, observable by students, and consistent with expected kinds of engagement at the middle school level. Participants responded to each item on a five-point scale (1 = *strongly disagree* to 5 = *strongly agree*). Higher scores on the student engagement scale indicate higher levels of engagement in school.

#### Fundamental Academic Behaviors

The Fundamental Academic Behaviors (FAB) scale was developed to capture students’ perceived attendance and schoolwork completion. These are the basic foundations necessary for school engagement because if students do not show up or do not complete schoolwork, opportunities for learning and more meaningful academic engagement are limited. The scale included four items, which are (a) I attended all classes, (b) I met all the deadlines I set for myself, (c) I met all my teachers’ deadlines, and (d) I completed all my assigned schoolwork. Participants responded to each item on a five-point Likert scale (1 = *strongly disagree* to 5 = *strongly agree*).

#### Daily School Aversion

The daily school aversion was assessed by two items about how students feel—“right now” about school (“hate school” and “tired of school”; Lewis et al., 2017) on a four-point Likert scale (1 = *no*; 2 = *not really*; 3 = *sort of*; and 4 = *yes*) for five days. The two items were first summed to produce a daily score out of 8, and daily school aversion scores over 5 days were averaged. Higher scores indicate higher levels of school aversion.

### Statistical Analyses

Descriptive statistics and reliabilities were conducted to gain a basic understanding among the major variables by using SPSS 22.0. The means, standard deviations, skewness, and kurtosis values for each item and variable were examined for abnormalities and outliers. To estimate the reliability of the major variables, McDonald’s Omega (ω) was calculated, which is considered a better estimator than coefficient alpha (Hayes & Coutts, 2020).

Confirmatory factor analyses (CFAs) were conducted first to evaluate the measurement models of the SRL practices and academic challenges. The Mplus 8.7 program (Muthén & Muthén, 1998–2012) was used with the robust maximum likelihood estimation procedure as the optimal approach for multivariate data sets with missing data. Five recommendations for assessing model fit were selected (Bentler, 1990; Bollen, 1989; Hu & Bentler, 1999; Steiger, 1990; Tucker & Lewis, 1973): (a) comparative fit index (CFI) values ≥.90, (b) the Tucker-Lewis index (TLI) ≥ .90, (c) the standardized root mean square residual (SRMR) ≤ .08, (d) 90% confidence interval (CI) accompanying the root mean square error of approximation (RMSEA) ≤ .06, and (e) the scaled chi-square (*p* > .05). The concurrent validity of SRL practices and academic challenges was examined by conducting correlations with school engagement, daily school aversion, and fundamental academic behaviors.

## Results

Table 1 presents descriptive statistics of the variables including the mean, standard deviation, skewness, and kurtosis. The mean scores for SRL practices were ranged from 3.20 (*SD* = .78) for time management to 3.66 (*SD* = .87) for behavioral engagement. The mean scores of academic challenges were ranged from 2.63 (*SD* = .82) for behavioral challenges to 3.00 (*SD* = .95) for initiating and sustaining challenges. Reliabilities for each of SRL-P and academic challenge subscales were examined prior to conducting factor analyses. McDonald’s Omegas for all subscales were shown to be acceptable (Table 1), which supported the appropriateness of factor analyses for these data.

**Table 1.** Descriptive Statistics for the Subscales of Self-Regulated Learning Practices and Academic Challenges.

	Mean	SD	Skewness		Kurtosis		Reliability
			Statistic	Std. Error	Statistic	Std. Error	
Self-regulated learning practices							
Task understanding	3.52	.63	−.20	.13	−.14	.26	.78
Goal management	3.29	.79	−.28	.13	−.18	.26	.89
Time management	3.20	.78	−.15	.13	.05	.26	.68
Motivation appraisal	3.37	.72	−.31	.13	.33	.26	.73
Adaptation	3.32	.68	−.15	.13	.28	.26	.83
Academic social engagement	3.63	.83	−.55	.13	.11	.26	.72
Monitoring	3.50	.79	−.30	.13	−.17	.26	.80
Task value	3.39	.73	−.38	.13	.11	.26	.61
Academic challenges							
Motivational	2.77	.98	.13	.13	−.60	.26	.79
Goal and time management	2.82	.92	.09	.13	−.59	.26	.83
Cognitive	2.73	.83	.03	.13	−.41	.26	.88
Social and emotional	2.71	.91	.05	.13	−.54	.26	.84
Metacognitive	2.70	.82	.08	.13	−.11	.26	.92
Initiating and sustaining	3.01	.92	.03	.13	−.49	.26	.79

**Figure 1.**
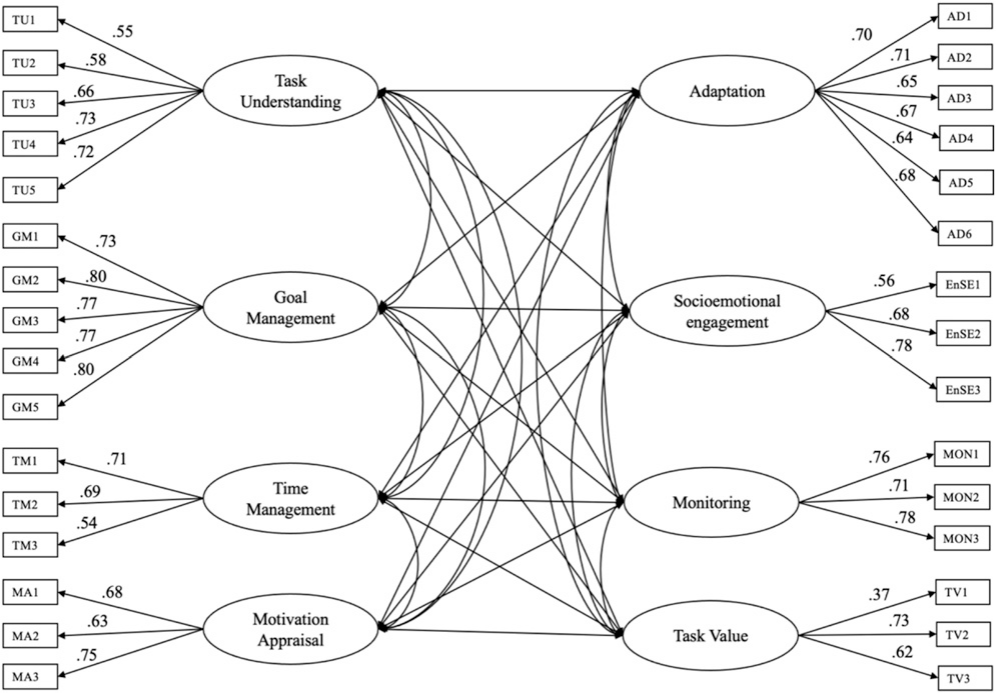
Factorial Structure of SRL Practices with Eight Latent Factors.

**Figure 2.**
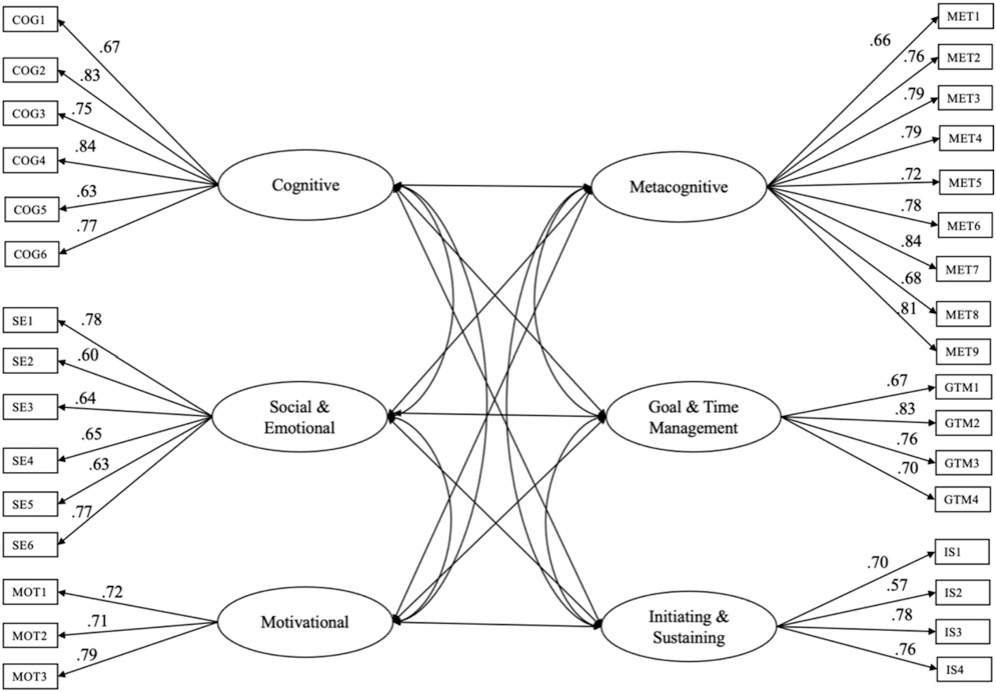
Factorial Structure of Academic Challenges with Six Latent Factors.

Confirmatory factor analyses (CFAs) were conducted to verify a priori factor structure of the SRL-P and SRL-C scales. CFA results revealed a good model fit for an 8-factor solution for the SRL-P scale (χ^2^ = 605.572, *df* = 406, *p* < .001, CFI = .95, TLI = .95, RMSEA = .038 [.031, .044], SRMR = .04). All items were loaded on their corresponding factors (Figure 1). CFA results also showed an adequate model fit for a 5-factor solution for the SRL-C scale (χ^2^ = 912.735, *df* = 419, *p* < .001, CFI = .91, TLI = .90, RMSEA = .058 [.053, .064], SRMR = .05). All items were loaded on their corresponding factors (Figure 2).

Correlational analyses were conducted to examine the concurrent validity of the SRL practices and academic challenges with student engagement, daily school aversion, and fundamental academic behavior (Table 2). We found that all types of SRL practices were significantly and negatively associated with all types of academic challenges. The results also showed that student engagement and fundamental academic behaviors were positively and strongly correlated with all types of SRL practices and negatively correlated with all types of academic challenges. Students’ daily school aversion was negatively correlated with all types of SRL practices and positively correlated with all types of academic challenges.

**Table 2.** Correlations Among Self-Regulated Learning Practices, Academic Challenges, School Engagement, Fundamental Academic Behaviors, and Daily School Aversion.

	TU	GM	TM	MA	AD	ASE	MO	TV	C-MOT	C-GTM	C-COG	C-SE	C-META	C-IS
TU	/													
GM	.72**	/												
TM	.63**	.76**	/											
MA	.74**	.73**	.66**	/										
AD	.75**	.74**	.67**	.76**	/									
ASE	.54**	.55**	.50**	.55**	.59**	/								
MO	.74**	.68**	.65**	.69**	.71**	.50**	/							
TV	.64**	.55**	.51**	.59**	.65**	.39**	.55**	/						
C-MOT	−.38**	−.40**	−.38**	−.34**	−.34**	−.33**	−.34**	−.20**	/					
C-BEH	−.37**	−.39**	−.44**	−.35**	−.31**	−.27**	−.33**	−.19**	.71**	/				
C-COG	−.40**	−.36**	−.37**	−.30**	−.30**	−.29**	−.36**	−.23**	.78**	.74**	/			
C-SE	−.30**	−.24**	−.25**	−.21**	−.26**	−.30**	−.25**	−.11*	.73**	.57**	.66**	/		
C-META	−.43**	−.39**	−.43**	−.35**	−.34**	−.32**	−.40**	−.27**	.80**	.82**	.85**	.70**	/	
C-IS	−.39**	−.41**	−.45**	−.32**	−.39**	−.37**	−.42**	−.24**	.70**	.71**	.65**	.62**	.76**	/
SE	.61**	.62**	.58**	.58**	.61**	.51**	.58**	.43**	−.50**	−.45**	−.46**	−.37**	−.51**	−.55**
FAB	.51**	.50**	.56**	.49**	.51**	.40**	.46**	.40**	−.37**	−.51**	−.34**	−.27**	−.42**	−.42**
DSA	−.38**	−.39**	−.38**	−.33**	−.38**	−.39**	−.35**	−.19**	.47**	.30**	.37**	.49**	.37**	.44**

*Note.* TU = task understanding, GM = goal management, TM = time management, MA = motivational appraisal, AD = adaptation, ASE = academic social engagement, MO = monitoring, TV = task value, C-MOT = motivation challenges, C-GTM = goal and time management challenges, C-COG = cognitive challenges, C-SE = social and emotional challenges, C-META = metacognitive challenges, C-IS = initiating and sustaining challenges, SE = school engagement, FAB = fundamental academic behaviors, DSA = daily school aversion.

*p < .05 and **p < .001.

## Discussion

The current study examined the psychometric adequacy of the SRL practices and academic challenges measures within the SRL-PSD instrument for adolescents. The concurrent validity of the SRL-PSD was also examined by correlating with well-established measures in relation to school engagement and feelings. This section discusses (a) major findings of examining the validities of SRL practices and academic challenges as well as the concurrent validity and (b) limitations and implications for the present study.

First, a good model fit for the eight-factor model of SRL practices was found, which supports the multi-component structure of self-regulatory practices and the use of these practices for adolescents in the middle school. This finding supports Hadwin et al.’s (2022) study by demonstrating that the SRL practices within the SRL-PSD measure have a good model fit for students in early adolescence and emerging adulthood. As Zeidner (2019) emphasizes, it is important to understand how the changes in SRL processes are affected by aging and maturation. Taken together, these results from the present and Hadwin et al.’s (2022) study correspond to another research by Schunk and Ertmer (1999) that addressed the developmental differences in acquiring and instructing self-regulatory skills. Based on the latent factor analysis, eight components of SRL practices, including task understanding, goal management, time management, task value, motivation appraisal, monitoring, adaptation, and academic social engagement, were strongly correlated. In line with SRL research (Losenno et al., 2020) for university students, these findings demonstrate that SRL practices in middle school are reciprocal and interactive. Overall, these results suggest that factors of SRL practices examined in this study are consistent with five aspects of SRL (i.e., task understanding, goal setting and planning, strategy enactment, adaptation, and monitoring and evaluation), which have been shown to be theoretically and empirically associated with student success outcome. These findings further build the foundation for future studies that aim to examine the longitudinal development of SRL practices over time.

In addition, psychometric properties of the academic challenges measure showed an adequate model fit for the six-factor model. This finding indicates appropriate use of the academic challenge measure for adolescents and align with Hadwin et al.’s (2022) study on the multiple components of academic challenges (i.e., metacognitive, cognitive, behavioral, motivational, and social-emotional). Also, we found that six types of academic challenges were strongly correlated, indicating that adolescents tend to experience more than one type of challenge simultaneously during their learning. Moreover, middle school students’ engagement in SRL practices was negatively and strongly associated with their reported academic challenges. This finding indicates that middle school students’ self-regulatory practices can potentially mitigate the academic challenges they experience. Indeed, other research has demonstrated that students who learn about SRL strategies can actively engage in academic tasks and achieve academic success (Dignath et al., 2008; Rosário et al., 2013; Zimmerman, 2002), compared to students lacking self-regulatory competencies who display maladaptive learning patterns in school (Rosário et al., 2013).

Furthermore, we evaluated the concurrent validity of the SRL practices and academic challenges measures by correlating them with three measures relevant to adolescents’ engagement in school, such as student engagement (Me and My School Revised), daily school aversion, and fundamental academic behaviors. The results showed that SRL practices and academic challenges were significantly associated with these measures. Particularly, SRL practices were found to be positively associated with students’ engagement in school and negatively associated with the school aversion; whereas academic challenges were found to be negatively associated with students’ engagement in school and positively associated with the school aversion. These findings suggest that middle school students who engaged in more SRL practices were more likely to engage in school activities and report more positive feelings toward school; oppositely, those who experienced more academic challenges were less likely to engage in school and more likely to develop school aversion. These results are consistent with the SRL literature indicating that actively engaging in SRL practices, including developing an adequate task understanding, strategically managing time, setting up a high-quality goal, and metacognitively adapting to various learning situations, motivates students to actively engage in school and feel positive toward school activities in terms of academic success. We postulate that students who proactively engage in their school activities tend to report more engagement in SRL practices and fewer academic challenges. This explanation is in line with the current literature that found school engagement contributes positively to learning outcomes, long-term involvement in schooling, and academic success (Heddy & Sinatra, 2013; Perry, 2008). Nevertheless, there is limited research examining whether middle school students’ engagement in school can predict their academic challenges, and how SRL plays a role in mitigating the effects of academic challenges on student engagement. Therefore, future research should explore the interactive relationships between student engagement, SRL, and academic challenges.

This study was the first to establish the validity and reliability of the SRL-PSD (Hadwin et al., 2022) comprising multiple components of SRL practices and academic challenges for middle school student populations. Our findings set an important stage for implementing SRL-PSD as a self-assessment for adolescents to evaluate their adaptive and maladaptive learning processes, thereby promoting the SRL capacity for adolescents who experience various transitions developmentally and academically. Since the SRL-PSD is designed as a therapeutic instrument to provide immediate feedback for learners to reflect on how they learn, having it validated for middle school students allows educators to incorporate it into their instructional design to teach SRL skills.

### Limitations and Implications

Although this study validates the SRL-PSD as a sound instrument for the assessment of SRL in middle school populations, several limitations should be considered alongside the results. The initial set of limitations includes sample size, participant demographics, and the measurement invariance of the SRL-PSD. Firstly, as this sample consisted of 358 middle school students from the Sooke School District of the province of BC, which comprises populations that mostly speak English as their heritage languages (Statistics Canada, 2023), Future studies should include larger and more diverse samples (e.g., ethnicity, culture, race, and language) to better represent Canadian middle schoolers. Recruitment from other Canadian provinces and territories would allow researchers to gather a larger sample and potentially shed light on the differences in middle schoolers’ SRL practices and academic challenges across Canada. As these differences could result from the differences in curriculum and middle school classification (e.g., middle school in Alberta ranges from grades 7 to 9, but middle school in BC goes from grades 6 to 8) across Canada, the inclusion of participants from a larger geographical area would increase the generalizability of results.

Secondly, as this study’s sample consisted of mostly white, educated, industrial, rich, and democratic (WEIRD; Henrich et al., 2010) participants, future research using the SRL-PSD should (1) include a diverse sample that is reflective of Canada’s multicultural population, (2) consider other Western cultures (e.g., United States and European countries) and non-Western cultures for its generalizability, and (3) evaluate its application to students with learning disabilities. To ensure the SRL-PSD is a reliable and valid measure of middle schoolers’ SRL practices and academic challenges, future research should establish the measurement comparability of the SRL-PSD across genders, races, ethnicities, grade levels, and populations with special learning needs.

The second set of limitations relates to the study design. That is, the cross-sectional nature of this study limits its ability to evaluate the developmental trajectory of adolescents’ SRL practices and challenges, and future research should consider examining the SRL-PSD longitudinally. For example, Zeidner (2019) argues that, to examine the complexity of SRL in student learning, experimental research should employ methods that capture students’ use of SRL processes over time. Hence, adopting a longitudinal design in future research using the SRL-PSD would allow for the examination of (1) the recursive and cyclical nature of middle schoolers’ SRL practices and academic challenges and (2) the associations between SRL practices and academic challenges in middle school. Most importantly, as longitudinal research can incorporate adolescents’ unique biological circumstances, it can also provide information on academic challenges adolescents experience and the SRL practices they employ during middle school. Therefore, if future longitudinal research employs both the SRL-PSD alongside other self-report measures in middle school populations, it could help identify (1) how adolescent development influences SRL and (2) how current SRL measures could be revised to reflect the unique biological changes that accompany adolescent development.

The final set of limitations relates to the predictive and concurrent validity of this study. As Hadwin et al. (2022) originally developed the SRL-PSD to address the SRL practices and academic challenges students experience in university, examining the predictive validity of the measure was straight forward using the standardized grading criteria and grade point average (GPA). However, as the K-9 curriculum in BC uses a provincial proficiency scale alongside written feedback, there is not a standardized grading criterion. Therefore, the lack of standardization constrained the ability to examine how the SRL-PSD could predict middle school students’ academic performance. Nevertheless, to ensure the SRL-PSD was a valid measure of middle schoolers’ SRL practises and academic challenges, this study conducted correlational analyses to examine the concurrent validity of the SRL-PSD by including three validated measures of middle schoolers’ academic engagement. Examining the concurrent validity of a newly developed measure by comparing it to a well-established measure is a reliable way of discerning new measure validity (Dörrenbächer et al., 2019). However, as this study did not examine the predictive power of SRL-practices and academic challenges, future research should consider other academic success outcomes (e.g., students’ retention and academic well-being) to examine the predictive validity of SRL practices and academic challenges measured by the SRL-PSD.

Lastly, we argue that using self-report measures of SRL provides opportunities for learners to gain insights into their perceptions and beliefs about their self-regulatory practices and develop a strong metacognitive awareness of their learning. Specifically, among various self-report measures of SRL (e.g., CP-SRLI, LASSI, and MSLQ; Pintrich et al., 1993; Weinstein et al., 1987; Vandevelde et al., 2013), the SRL-PSD instrument provides learners with individualized reports. These reports encourage them to consider, critique, discuss, and reflect on their engagement in SRL practices and academic challenges in terms of their learning strengths and weakness. These insights can serve as a valuable steppingstone for the design of SRL interventions and allow learners to develop the metacognitive awareness (Hadwin & Winne, 2012). An SRL measure such as this will provide students, teachers, parents, and researchers with aa timely snapshot of SRL practices and academic challenges.

In essence, SRL is a set of dynamic events (Butler & Cartier, 2017) unfolding over time through different contexts (McCardle & Hadwin, 2015). Therefore, to capture this in adolescent populations, it is important to incorporate SRL self-report measures (e.g., the SRL-PSD instrument) with event measures, such as trace logs of learners’ SRL processes (Winne and Nesbit, 2010), think-aloud methods (Azevedo et al., 2010), and intelligent tutoring systems (Azevedo et al., 2022). Combining the SRL-PSD alongside newly developed technologies for measuring SRL that provide observational data of students’ learning process (i.e., MetaTutor; Azevendo et al., 2022) could further our understanding of students’ SRL and, hopefully, provide teachers with tools needed to lessen students’ academic challenges and increase engagement with effective SRL practices.

## Data Availability

The data that support the findings of this study are available from the corresponding author upon reasonable request. The data are not publicly available due to confidentiality restrictions.*
